# Acids in Digestion

**Published:** 1887-08

**Authors:** 


### Acids in Digestion.

Hydrochloric and lactic acids are found in the healthy stomach within half-an-hour after taking food. Under an exclusive meat diet, hydrochloric acid is found *alone.* Under a mixed diet, hydrochloric, lactic, and volatile acids are found, in quantities varying according to the length of time the food remains in the stomach. In fever and in anemia, the hydrochloric acid is usually wanting. Small repeated doses of hydrochloric acid in warm water are exceedingly useful in dyspepsia and other gastric troubles. As a rule it is best taken after each meal. The acid is both an antiseptic and an anti-ferment. It forms the basis of many vaunted nostrums for "indigestion;" it is certainly efficacious in many forms of dyspepsia, as pointed out by Trousseau years ago.

